# Curcumin protects mice from *Staphylococcus aureus* pneumonia by interfering with the self-assembly process of α-hemolysin

**DOI:** 10.1038/srep28254

**Published:** 2016-06-27

**Authors:** Jianfeng Wang, Xuan Zhou, Wenhua Li, Xuming Deng, Yanhong Deng, Xiaodi Niu

**Affiliations:** 1 Key Laboratory of Zoonosis, Ministry of Education, Institute of Zoonosis, and Department of Food Quality and Safety, College of Veterinary Medicine, Jilin University, Changchun, China

## Abstract

α-hemolysin (Hla) is a self-assembling extracellular protein secreted as a soluble monomer by most *Staphylococcus aureus* strains and is an essential virulence factor for the pathogenesis of various *S. aureus* infections. Here, we show that curcumin (CUR), a natural compound with weak anti-*S. aureus* activity, can inhibit the hemolysis induced by Hla. Molecular dynamics simulations, free energy calculations, and mutagenesis assays were further employed for the Hla-CUR complex to determine the mechanism of such inhibition. The analysis of this combined approach indicated that the direct binding CUR to Hla blocks the conformational transition of Hla from the monomer to the oligomer, leading to an inhibition of Hla hemolytic activity. We also found that the addition of CUR significantly attenuated Hla-mediated injury of human alveolar cell (A549) co-cultured with *S. aureus*. The *in vivo* data further demonstrated that treatment with CUR protects mice from pneumonia caused by *S. aureus*, including methicillin-resistant strains (MRSA). These findings suggest that CUR inhibits the pore-forming activity of Hla through a novel mechanism, which would pave the way for the development of new and more effective antibacterial agents to combat *S. aureus* pneumonia.

*Staphylococcus aureus* is a major community- and hospital-acquired pathogen that causes a broad spectrum of diseases, ranging from soft-tissue infections to life threatening deep tissue damage, and even more invasive diseases, such as osteomyelitis, endocarditis and pneumonia^1^. Among these infections, *S. aureus* pneumonia is considered one of the most prevalent and serious infections, with significant morbidity and mortality. The gradually increasing incidence of antibiotic resistant *S. aureus* strains has created an enormous burden on both healthcare and community settings in many countries^2^. Over the past few years, methicillin-resistant *S. aureus* (MRSA) has become a worldwide challenge to human and animal health, accounting for more than 50% of all staphylococcal pneumonia isolates. Along with the multi-drug resistance phenomenon, shortcomings associated with the limited number of agents currently approved for MRSA infection make research into novel treatment options against staphylococcal infections a priority. Recently, many studies have reported that targeting bacterial virulence factors by small molecules could attenuates the pathogensis of bacteria-mediated disease *in vitro* and *in vivo*, suggesting a promising therapeutic strategy for *S. aureus* infection^3^,^4^,^5^,^6^.

*S. aureus* produces a diverse array of virulence factors contributing to its pathogenesis, including exotoxin and surface-associated proteins. The virulence factor α-hemolysin (Hla; encoded by the *hla* gene) is a 33.2 kDa pore-forming hemolytic toxin which is produced by most pathogenic strains of *S. aureus*^7^. The toxin is known to cause the lysis of numerous types of mammalian cells, including erythrocytes, lymphocytes, alveolar epithelial cells, macrophages, and monocytes. Hla, a water-soluble monomer secreted by *S. aureus*, is capable of binding to the membrane of the target cell membrane and oligomerizing into a 232.4 kDa membrane-inserted heptamer that penetrates the cell membrane, which ultimately results in cell damage and death^8^. There is increasing evidence implicating Hla in the pathogenesis of human disease, especially in staphylococcal pneumonia. *S. aureus* mutant strains lacking *hla* exhibits significant loss of virulence in animal models of pneumonia^9^,^10^. Additionally, according to Bubeck *et al*. and Montgomery *et al*., the hypervirulent phenotype of current epidemic USA 300 isolates of MRSA is largely dependent on increased expression of virulence factors such as Hla and Panton-Valentine leukocidin^9^,^11^,^12^. Consequently, there is considerable interest in the development of small molecule inhibitors or antibodies for the treatment of Hla-induced diseases.

Curcumin (CUR) (Fig. 1a), an important active phenolic compound derived from the rhizome of *Curcuma longa* (Jianghuang), has outstanding anti-inflammation, anti-tumor and neuroprotective effects^13^. CUR has been used to treat cardiovascular disease, inflammatory bowel disease and cancer^14^,^15^,^16^,^17^. In our previous study, we reported that the direct engagement of some natural flavonoid compounds to Hla could neutralize the hemolytic activity of Hla by restraining the conformation change of the binding cavities^5^,^18^. In the current study, we found that CUR could inhibit Hla-mediated hemolysis at low concentrations. Molecular dynamics simulations, free energy calculations, and mutagenesis assays were further employed for the Hla-CUR complex to determine the mechanism of such inhibition. With these approaches, a new mechanism of inhibition by CUR was obtained compared with previous compounds. We have identified CUR, which binds to the new active sites (residues Gln89, Pro91, Thr161, Asp162, and Lys163) of Hla, as a novel potential antagonist of the Hla self-assembly process. Moreover, treatment with CUR provided a robust protective effect against *S. aureus* pneumonia in a mouse model of infection.

## Results

### CUR inhibits the hemolytic activity of Hla

**Figure 1:**
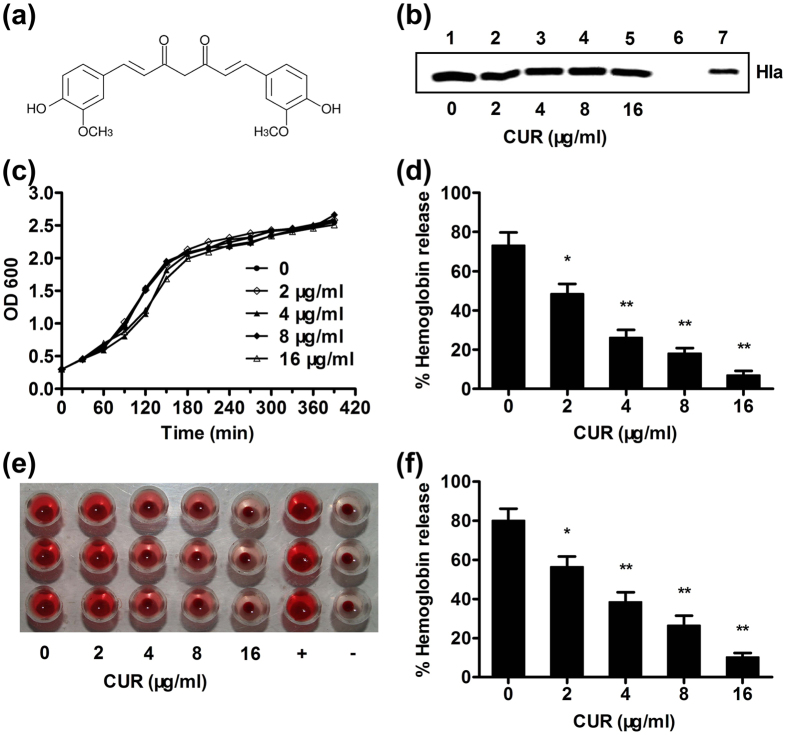
Curcumin (CUR) inhibits Hla-mediated hemolysis. (**a**) Chemical structure of CUR. (**b**) Demonstrating Hla expression in culture supernatants by Western blot. *S. aureus* strains 8325-4 (lanes 1–5) and DU 1090 (lane 6) were cultured with the indicated concentrations of CUR. Lane 7, 10 ng purified Hla. (**c**) Growth rate of *S. aureus* 8325-4 grown with various concentrations of CUR. (**d**) Hemolytic activity of culture supernatant of *S. aureus* 8325-4 treated with or without CUR. (**e**,**f**) Inhibition of purified Hla-mediated hemolysis by CUR. Bars represent the standard deviation of three independent experiments (*indicates *P* < 0.05 and **indicates *P* < 0.01; two tailed Student’s t-test).

In this study, we found that the MICs of CUR for both of the tested *S. aureus* strains were 256 μg/ml, indicating that CUR has weak anti-*S. aureus* activity; the growth curves for *S. aureus* 8325-4 cultured with 2 to 16 μg/ml of CUR are presented in Fig. 1c and indicate that these concentrations of CUR do not influence the growth of *S. aureus*. We also demonstrated that CUR was able to significantly inhibit the hemolytic activity of *S. aureus* culture supernatant (Fig. 1d) without affecting Hla expression by *S. aureus* (Fig. 1b). Moreover, purified Hla-mediated hemolysis was concentration-dependently attenuated by CUR (Fig. 1e,f), which is completely different from natural compounds that reduce the hemolytic activity by inhibiting the expression of *S. aureus* Hla. Thus, it seems reasonable to assume that CUR interacts directly with the Hla protein.

### CUR protects A549 cells from Hla-mediated cell injury

**Figure 2:**
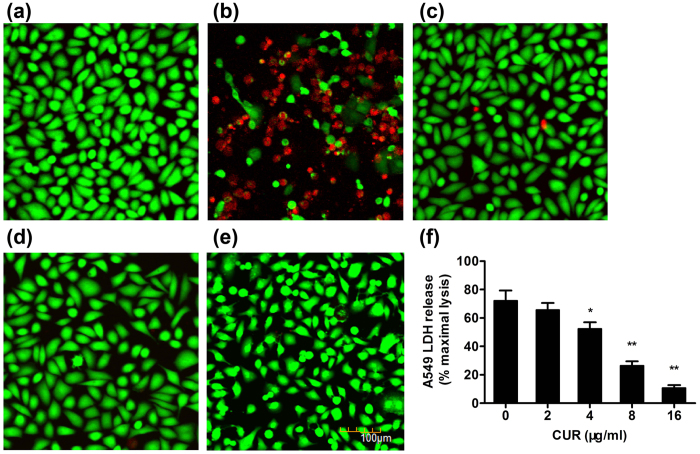
Alleviation of *S. aureus* Hla-mediated human alveolar epithelial cell injury by CUR. Human alveolar epithelial (A549) cells stained with live (green)/dead (red) reagent was imaged using confocal laser scanning microscopy following 7 h incubation with *S. aureus* 8325-4 or DU 1090. (**a**) Uninfected cells; cells infected with *S. aureus* 8325-4 in the absence of CUR (**b**) or the presence of 16 μg/ml of CUR (**c**); cells co-cultured with DU 1090 (**d**) or 16 μg/ml of CUR (**e**); (**f**) cell Cytotoxicity induced by *S. aureus* 8325-4 in the presence of indicated concentrations of CUR was evaluated by LDH release assay. Bars represent the standard deviation (*indicates *P* < 0.05 and **indicates *P* < 0.01; two tailed Student’s t-test).

A549 cells have been widely used in pulmonary disease models, and the significance of Hla in *S. aureus*-mediated human alveolar cell injury, as the mutant lacking Hla do not cause cellular injury, has been described in a previous study^19^. Therefore, based on the above mentioned findings, we further evaluated the ability of CUR to protect A549 cells from Hla-mediated injury in the co-culture system of A549 cells and the *S. aureus*. As shown in Fig. 2b, A549 cell injury in samples infected with *S. aureus* 8325-4 was apparent, as reflected by an increase in the amount of red fluorescence and morphological changes in the live cells. However, significantly lower number of red fluorescent dead cells were observed in the infected sampled in the presence of 16 μg/ml of CUR resulted in (Fig. 2c). Consistent with the previous study, the Hla-deficient mutant *S. aureus* DU 1090 did not cause cell death (Fig. 2d). Importantly, CUR did not affect the viability of A549 cells and showed no toxic effect on the cells at a concentration of 16 μg/ml (Fig. 2e). Moreover, the level of lactate dehydrogenase (LDH) released into culture supernatant was determined to quantify cellular injury and the result indicates that CUR, at the concentrations of 2 to 16 μg/ml, has the ability to protect A549 cells from injury in a dose-dependent manner (Fig. 2f). Collectively, these data reveal the *in vitro* efficacy of CUR in protecting human alveolar epithelial cells from cell death induced by Hla.

### CUR protects mice from *S. aureus* pneumonia

**Figure 3:**
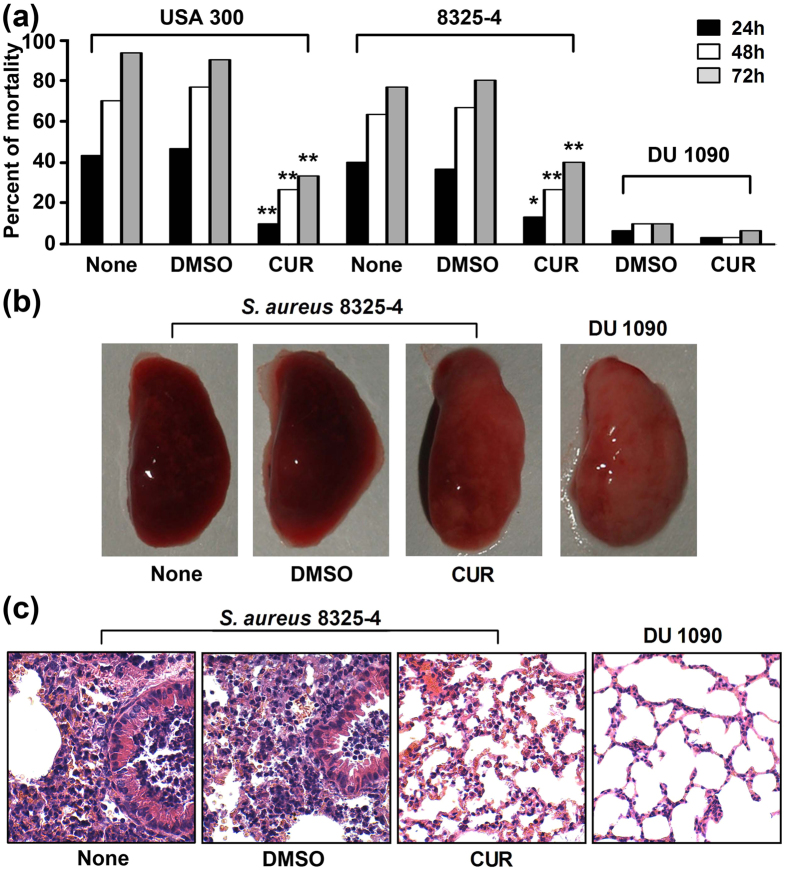
CUR inhibits *S. aureus* virulence in a mice model of pneumonia. Mice were nasally infected with 2 × 10^8^ CFUs of *S. aureus* 8325-4, DU 1090 or USA 300. (**a**) Mortality of infected mice without any treatment or treated with CUR or DMSO. *indicates *P* < 0.05, and **indicates *P* < 0.01 (two tailed Student’s t-test). Gross pathological changes (**b**) and histopathology (**c**) of lung samples from infected mice.

The *in vitro* protective effect against Hla-mediated injury of human lung cells by CUR prompt us to further analyzed whether similar *in vivo* protective effects occurs in a mouse infection model. Mice were infected intranasally with 4 × 10^8^ CFUs of *S. aureus* 8325-4 or USA 300. Following treatment with CUR, mortality of infected mice was monitored over 72 h. Negative controls were given an Hla-deficient strain DU 1090, while positive controls were treated with or without DMSO. As shown in Fig. 3a, administration of 100 mg/kg of CUR significantly protected mice from *S. aureus* pneumonia at 24, 48, and 72 h; however, a low mortality occurred in mice infected with *S. aureus* DU 1090, which was similar to previous reports from other groups. Furthermore, no significant difference was observed between the groups treatment with or without the vehicle DMSO, indicating that DMSO has no toxic effect on the infected mice (Fig. 3a). Interestingly, we found that CUR was also effective in mice infected by the highly virulent MRSA strain USA 300, which is a clinically relevant isolate causing a large number of cases of severe staphylococcal pneumonia. Thus, interference with Hla by CUR could significantly inhibit both methicillin-sensitive and methicillin-resistant *S. aureus* virulence, indicating that the therapeutic effect of antiinfective therapy with Hla inhibitor for *S. aureus* infection is associated with the role of Hla for bacterial pathogenicity but not the antimicrobial resistance. Moreover, the pathological manifestations were determined by histopathologic analysis of lungs from infected mice treated with CUR or DMSO to evaluate the impact of CUR treatment. Only focal infection with a reduction in the dense and red appearance was observed for the lung tissue of infected mice that received CUR (Fig. 3b), while the majority of the airspace was obliterated by inflammatory cell infiltrates in the samples treated with DMSO (Fig. 3c). Remarkably, CUR treatment resulted in a significant alleviation of pulmonary inflammation, as indicated by less accumulation of cellular infiltrates in the alveolar space. Consistent with earlier results, the Hla mutant strain DU 1090 did not cause visible injury to the lung tissue. Taken together, our results establish that CUR treatment confers systematic protection against *S. aureus* pneumonia in a mouse model of infection.

### Determination of the binding mode of Hla with CUR

**Figure 4:**
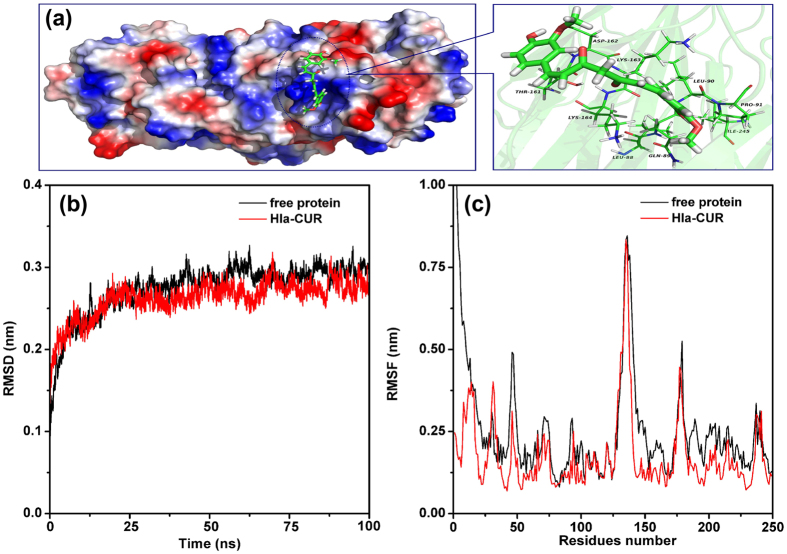
The interactions between CUR and Hla predicted by modeling. (**a**) 3D structure of the complex of Hla with CUR by MD simulation. (**b**) The RMSD of the backbone atoms of the protein in the free Hla or Hla-CUR complex system. (**c**) RMSF of all the residues in the Hla protein (black line), and in the Hla-CUR complex (red line). The residues 80–100 and 150–175 in the binding cavity of Hla are highlighted with gray bars.

Due to the direct interaction between CUR and Hla, we next explored the mechanism of action of CUR molecular modeling. Then, the binding mode of CUR with Hla was determined based on the 100-ns molecular dynamics simulations. It was shown that CUR can bind to Hla via hydrogen bonding and van der Waal interaction. In the complex system, CUR can bind to the “stem” region of Hla (Fig. 4a). As reported in the previous literure^8^,^20^, the stem region is the key position, which participated in the self-assembly of Hla. To indicate the convergence of protein structures, the RMSD values of the protein in the whole systems as a function of time is shown in Fig. 4b. The backbone RMSD of protein can reach the equilibrium after ~20 ns of simulation, as shown in Fig. 4b, which provides assurance that the final 50 ns of the simulation are suitable for analysis.

The 3D structure of CUR with Hla was shown in Fig. 4a. In detail, the Pro91 and Gln89 anchor the 4H-chromen-4-one moiety of CUR. As shown in Fig. 4a, the 4H-chromen-4-one moiety of CUR are close to the pyrrolidine ring of Pro91 and the propionamide chain of Gln89, indicating that Pro91 and Gln89 have strong van der Waal interactions with CUR. On the other hand, Thr161, Asp162 and Lys163 also have the strong interaction with CUR. Further, the side chains of Thr161 and Asp162 are close to the benzene ring of CUR, indicating that a strong van der Waals interaction between Thr161, Asp162 and CUR existed (Fig. 4a).

In addition, the root-mean-square fluctuation (RMSF) is used to provide higher resolution details of residue fluctuations. The fluctuation patterns of the complexes and the free protein in a specific region of Hla are different at the final 50 ns of the simulation (Fig. 4c). In complex system, the residues in the binding site become bound due to the binding with inhibitors, which leads to the lower degree of flexibility (the RMSF values less than 0.375 nm). However, the RMSF value in the free protein system is exactly higher than that of complex system. Taken together, the above results show that the residues Gln89/Leu90/Pro91/Thr161/Asp162/Lys163 are the crucial binding sites for the stabilization of the Hla binding cavity in this complex.

### Identification of the binding site in the Hla-CUR complex

**Figure 5:**
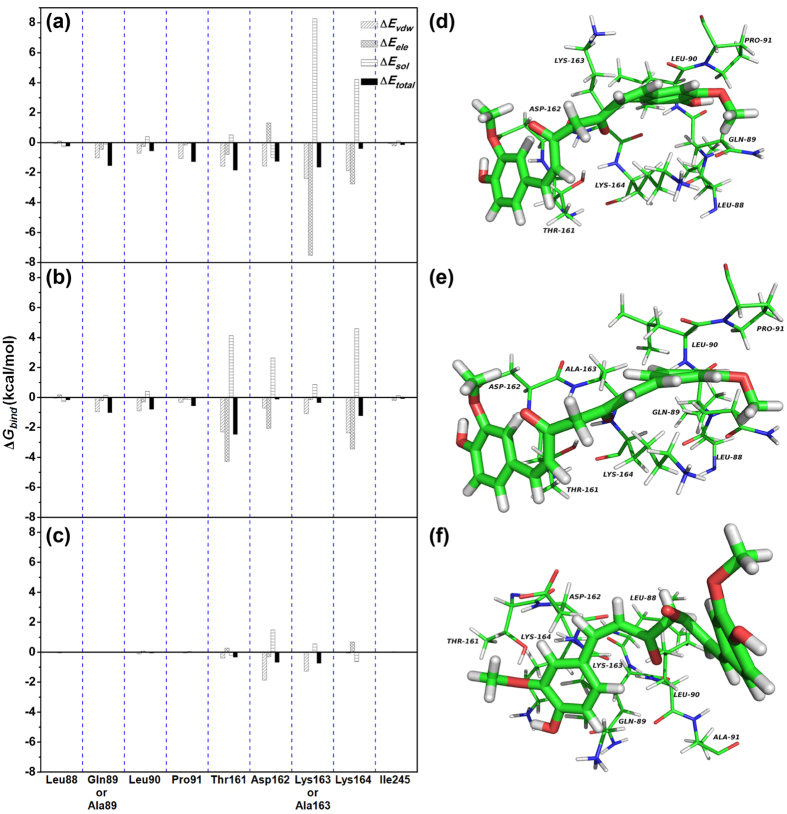
Decomposition of the binding energy on a per-residue basis in the Hla-CUR complex. (**a**) WT-Hla-CUR complex; (**b**) K163A-CUR complex; (**c**) Q89A-CUR complex. The 3D structures of CUR with the WT-Hla (**d**), K163A (**e**), and Q89A (**f**) are shown with labels for key residues.

To confirm the above hypothesis about the binding sites in this complex, the calculation of the binding free energy between the residues surrounding the binding site and the inhibitor were performed by using the MM-GBSA method^21^,^22^ (Fig. 5). As shown in Fig. 5a, an appreciable van der Waals (*ΔE*_vdw_) contribution of Gln89 exists, with a *ΔE*_vdw_ of ~−1.10 kcal/mol due to the close of the side chain of Gln89 with the 4H-chromen-4-one moiety of CUR. Furthermore, Pro91 also has an appreciable van der Waals interaction with the inhibitor because of the close proximity between the pyrrolidine ring of residue Pro91 and the 4H-chromen-4-one moiety of CUR. In addition, residue Thr161 and Asp162 also have the strong contributions to the binding with the inhibitor, with the *ΔE*_vdw_ of −1.87 and −1.89 kcal/mol (Fig. 5a). Interestingly, residue Lys163 has an obvious electrostatic term (~−7.2 kcal/mol) and van der Waals term (~−2.2 kcal/mol), while solvation (*ΔE*_sol_) shows unfavorable contributions at ~8.1 kcal/mol; consequently, the total energy contributions of Lys163 is strong (~−1.72 kcal/mol). These results are consistent with the data shown in Fig. 4, which indicates that the residues of Gln89/Pro91/Thr161/Asp162/Lys163 could be the key binding sites in the complex.

Furthermore, the total binding free energy between Hla and CUR was calculated to verify the accuracy of the above hypothesis by using the MM-GBSA approach (see Supplementary Table S1). *ΔG*_nonpolar_ + *ΔE*_vdw_ is the primary contributor to the total free energy of the complex, with a value of −24.22 kcal/mol. In contrast, *ΔG*_ele, sol_ + *ΔE*_ele_ has a minor contribution of −15.96 kcal/mol. To verify the accuracy of the binding site in the Hla-CUR complex, the molecular modelings were performed for the two mutants, K163A-Hla and Q89A-Hla, with the same procedure. As shown in Supplementary Table S1, the binding energy of mutants were exactly weaker than that of WT-Hla with estimated *ΔG*_bind_ values of −10.28, −7.43 kcal/mol for K163A-CUR, Q89A-CUR, respectively. As shown in Fig. 5d–f, CUR binds to K163A-Hla, Q89A-Hla and WT-Hla in a similar manner, which was confirmed by pair interaction decomposition of the free energy (Fig. 5b,c).

**Figure 6:**
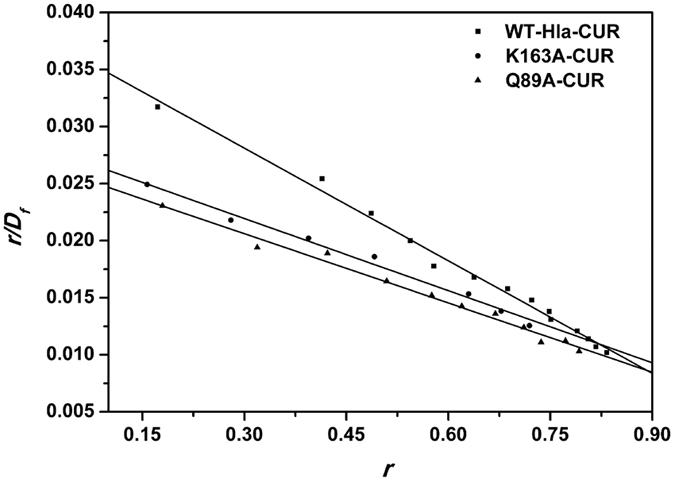
The Scatchard plots of *r/D*_*f*_ vs. *r* for CUR binding to WT-Hla, K163A, and Q89A.

To verify the result of MD simulations, the interactions between CUR and WT-Hla, K163A-Hla, Q89A-Hla were investigated by fluorescence quenching. The proteins were used as the fluorophore and CUR was used as the quencher. Commonly, fluorescence quenching can be described by the following Scatchard equation^23^,^24^: *r*/*D*_*f*_ = *nK* − *rK*, where *r* is the ligand amount of substance per mole of protein binding (*r* ≈ *ΔF/F*_*0*_), *D*_*f*_ is the free concentration of CUR, *K* is the binding constant and *n* is the number of binding sites. In this system, due to the CUR concentration being far greater than the concentration of protein; the *D*_*f*_ is replaced by the total CUR concentration, *Q*. According to experimental results, the linear fitting plots of *r*/*D*_*f*_ vs. *r* between CUR and WT-Hla, K163A-Hla, Q89A-Hla can be made in Fig. 6 and based on the plots the values of *K* and *n* can be obtained and are shown in Supplementary Table S2. The value of *n* is nearly 1, which indicates that in the binding reaction, the molar ratio of CUR to protein is 1:1.

According to data shown in Supplementary Table S2, the binding constants of the interaction between CUR and proteins decrease in the following order: WT-Hla > K163A-Hla > Q89A-Hla at 300 K, which indicates that the binding of WT-Hla with CUR is stronger than those of mutants. The experimental results are mostly consistent with the theoretical results (see Supplementary Table S2). It is believed that the 3D structure of Hla-CUR complex is reliable by MD simulation.

### The conformation change of the “stem” in the complex

**Figure 7:**
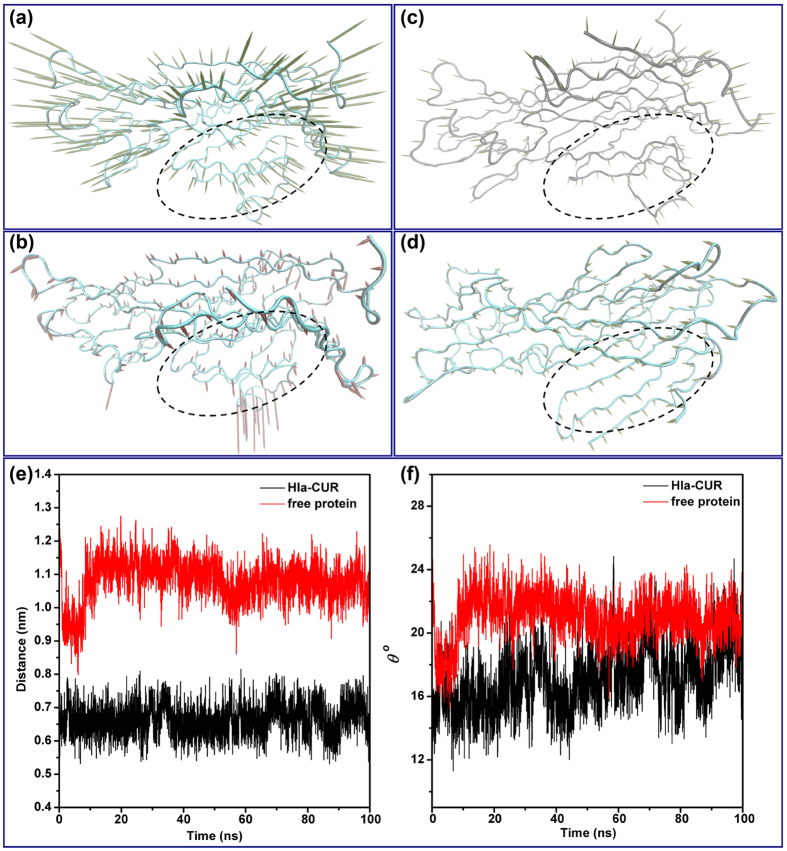
Analysis of the essential dynamics for Hla. The first two principal components in free protein (PC1: **a**, PC2: **b**) and complex (PC1: **c**, PC2: **d**) are shown by cones on the alpha carbon atoms. The magnitude of the motion was represented by the length of the cones. The “stem” region was surrounded by the dotted line. (**e**) The distances between the Cα atoms of Ser99 and Gly122 in free Hla (black line) and the complex of Hla-CUR (red line) during the MD simulation were shown. (**f**) The angle between Ser99, Thr109 and Gly122 in the complex (red line) and free protein (black line) during the MD simulation were shown.

In this work, our data suggested that CUR could inhibit the hemolytic activity of Hla and bind to Hla in the “stem” cavity, a critical position for Hla hemolytic activity, by MD simulation. Through the analysis of the cavity dynamics, we found that the conformation of the binding cavity, “stem”, in the complex system is completely different with that in the free protein system. As shown in Fig. 7e, the average distance between the C_α_ of Ser99 and the C_α_ of Gly122 is 0.67 nm (ranged from 0.60 to 0.75 nm) during the simulation time. However, in the free protein system, average distance between the same defined points was 1.06 nm (ranged from 1.00–1.20 nm) during the simulation time. Furthermore, the angle between Ser99, Thr109 and Gly122 in the complex and free protein were calculated. As shown in Fig. 7f, the average angle (22 degree) in the free protein was obviously bigger than that of the complex system (18 degree). Based on the above results, the “stem” domain of Hla was constrained due to the binding of CUR with Hla.

Subsequently, to identify the most significant motions of the protein in the complex system or free protein system, the principal component analysis (PCA) of the MD trajectory of the free Hla and Hla-CUR complex was calculated. In the free protein system, the first two principal components account for 88.71% and 11.22% of the overall motion. As shown in Fig. 7a, to the “stem” domain in the Hla, an obviously extended motion exists. This motion is the critical process for the conformational transition of Hla from monomer to the oligomer. As shown in Fig. 7b, a slight vibration of the protein exactly corresponds to the PC2. On the other hand, for the Hla-CUR complex system, the first two principal components account for 85.43% and 12.71% of the overall motion. As shown in Fig. 7c, the mostly motions of PC1 is similar to those of the free protein system except for the motion of the “stem” domain. Obviously, the motion of the “stem” domain is weaker than that of the free protein, which is due to the binding of CUR with Hla in the binding cavity. This result is consistent with the previous data, and we can believe that the motion of the “stem” domain is restricted due to the binding of CUR with Hla. Furthermore, a novel inhibition mechanism is developed: the conformation transition of the “stem” domain from monomer to the oligomer is blocked by the binding of CUR, which lead to the lytic loss of Hla.

**Figure 8:**
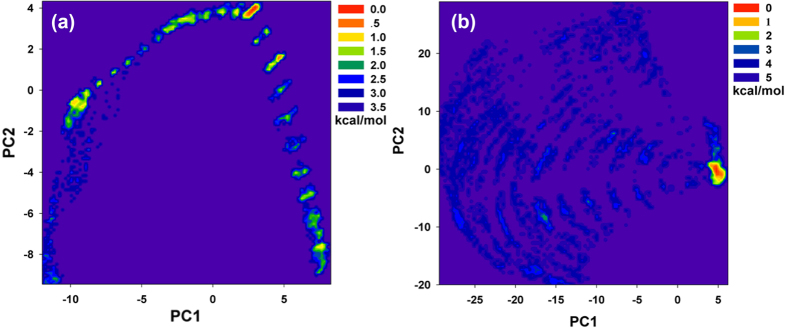
The energy landscape of Hla. The 2D contour map of the energy surface for the protein in the free Hla system (**a**) and the complex system (**b**). The first two principal components were used to the coordinates.

To confirm the hypothesis, the free energy landscapes of the conformational transition of Hla in the complex or free protein were analysis. As shown in Fig. 8a, the conformations of the free protein distribute more loosely, and show 5 deep wells at (−10, −1.8), (3.7, −2.5), (4, 2.5), (2, 5), and (−6, 7.5), which indicates that the conformations of the free protein are more slack and can meet the requirements for the conformational transition of Hla to transition from monomer to the oligomer (including the “stem” domain). However, for the Hla-CUR complex (Fig. 8b), the conformations of the protein distribute more compactly, and show only one deep well at (5, 0), which indicates that the conformations of the protein in the complex are restrained by the binding of CUR with the “stem” domain. These results are agreement with the above mentioned hypothesis.

**Figure 9:**
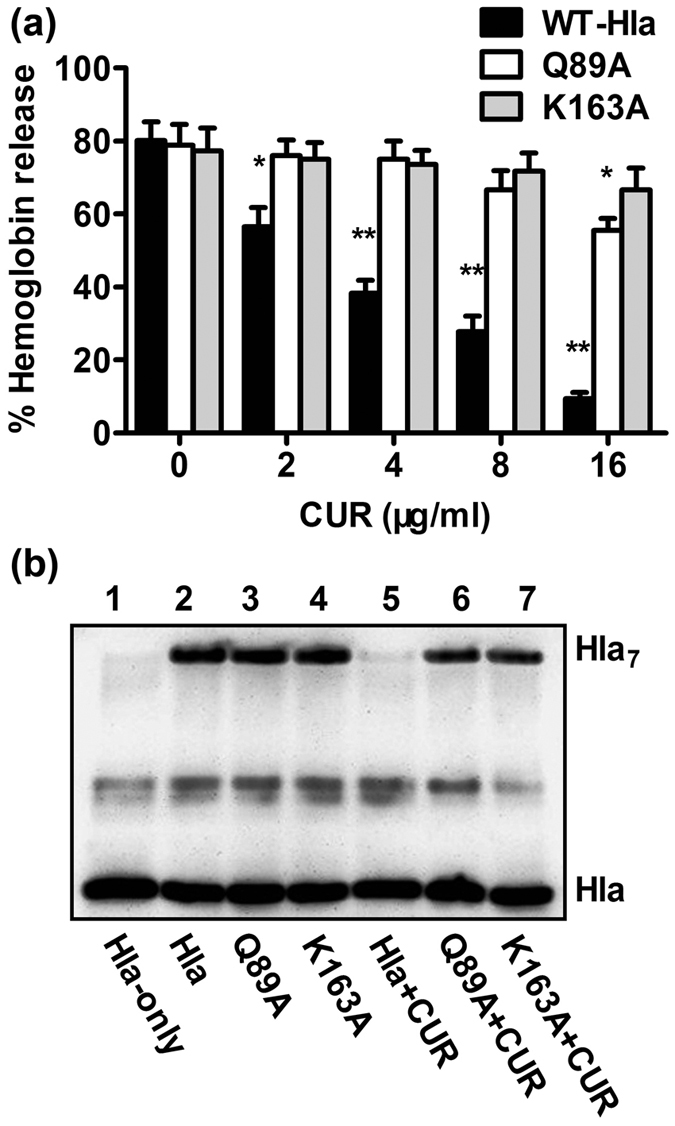
CUR interferes with the oligomerization of Hla induced by deoxycholate. (**a**) The influence of CUR on the hemolysis induced by Hla and its two mutants (*indicates *P* < 0.05 and **indicates *P* < 0.01; two tailed Student’s t-test). (**b**) CUR reduces the oligomer of Hla. The oligomerization of Hla and its two mutants was determined by western blot analysis after treated with 5 mM deoxycholate in the presence or absence of CUR. Lane 1, WT-Hla without 5 mM deoxycholate; lane 2–4, WT-Hla, Q89A-Hla and K163A-Hla treated with 5 mM deoxycholate, respectively; lane 5–7, WT-Hla, Q89A-Hla and K163A-Hla treated with 5 mM deoxycholate in the presence of 16 μg/ml of CUR, respectively.

To further confirm this hypothesis, a deoxycholate-induced oligomeriazation assay was employed and the results showed that the assembly of the SDS stable oligomer, Hla_7_, was not affected by site-directed mutagenesis of K163A and Q89A (Fig. 9). However, the addition of 16 μg/ml CUR significantly inhibited the formation of Hla_7_, which is in good agreement with the calculated results. Further, the inhibitory effect of CUR against Hla_7_ formation of either of the two mutants was decreased due to the weaker affinities of the ligand binding with Hla in the protein-ligand complex (Fig. 9b). Taken together, our data established that the conformational change from monomer to heptamer of Hla was blocked by the binding of CUR to the “stem” domain, which inhibits the lytic activity of Hla (Fig. 9a).

## Discussion

Unquestionably, the global use of antibiotic agents has improved the quality and length of life for countless people since their earliest discovery. It was once thought that most bacterial infectious diseases had been conquered due to the use of antibiotics. However, such an earlier optimism is now challenged by the increasing emergence of antibiotic-resistant bacteria and unavailability of effective antibiotics. In *S. aureus* infections, the incidence of staphylococcal infection associated with MRSA that cause invasive infections, such as necrotising pneumonia with high levels of morbidity and mortality, are continuing to increasing, while the antibacterial agents available today against this pathogenic bacterium are extremely limited^25^,^26^. Currently, vancomycin and linezolid are recommended as empirically proven and definitive therapies for treating MRSA pneumonia^27^. Unfortunately, in some cases, no positive effect was observed for the treatment of *S. aureus* infection in patients even with appropriate doses of antibiotics. *S. aureus* pneumonia has been considered one of the most prominent diseases and a worldwide threat to public health. Therefore, new therapeutic strategies and antimicrobial agents for infections caused by *S. aureus* are greatly needed. Interference with bacterial virulence has emerged as effective and promising strategy in meeting the challenge^3^. Although the traditional strategies are aimed at cellular viability (bacteriocidal and bacteriostatic activity) associated with highly effective, these modes of action results in the growth of drug-resistant strains. In contrast, antivirulence strategies provide promising opportunities for abating pathogenicity and its consequences, without directly killing the target bacteria, thus presumably applying a milder selective pressure for the development of resistance^3^.

*S. aureus*, like other Gram-positive bacteria, secretes a great deal of extracellular virulence factors that contribute to its pathogenicity, such as Hla, enterotoxins and cell wall-associated proteins^28^. Moreover, previous studies have reported that high expression of virulence factors in MRSA contributes to a hypervirulent phenotype of this microorganism, resulting in more severe and widespread disease^29^,^30^. In *S. aureus*, the significance of Hla in the pathogenesis of infection has been well established and demonstrated in multiple animal infection models including pneumonia, meningitis, mastitis and septic arthritis^10^,^31^. Based on these findings, Hla may represent a promising antivirulence target for the development of novel agents for *S. aureus*. To date, some successful examples of strategies aimed at attenuating the detrimental function of Hla in an animal model of pneumonia have already been reported. Wardenburg and colleagues have established the therapeutic value of treating *S. aureus* pneumonia by either Hla antibodies or a modified β-cyclodextrin compound, an Hla inhibitor by blocking the heptameric pore^4^,^19^,^32^. Moreover, our previous studies have also demonstrated that some natural compounds can attenuate *S. aureus* virulence in a mouse pneumonia model via inhibiting Hla expression or the pore-forming activity of this cytotoxic protein^5^,^18^,^33^.

In the present study, we found that CUR, a natural compound with weak anti-*S. aureus* activity, can inhibit the hemolytic activity of Hla directly without affecting the production of Hla or the growth of this organism. To insight into the mechanism of such inhibition, complementary methods, including molecular dynamics simulations, site-specific mutagenesis and a fluorescence-quenching method, were used for the complex system. Through these approaches, a possible inhibition mechanism was proposed: due to the binding of CUR to the “stem” region of Hla, the concomitant change in conformation of the “stem” region was restricted, leading to the inhibition of the self-assembly of the heptameric transmembrane pore, which decreases the biological activity of Hla for cell lysis. This novel inhibition mechanism could facilitate the development of new and more effective antibacterial agents. Although antibodies by neutralizing Hla activity controlled *S. aureus* virulence, CUR should be more advantageous than toxin-specific antibodies due to its lower chance of contamination, lower likelihood of triggering an immune reaction and lower cost in production and maintenance. Additionally, CUR is a multi-targeting molecule that has been shown to markedly attenuate *S. aureus*-induced pneumonia, barrier disruption, lung edema, and vascular leakage through multiple pathways including inhibiting NF-κB-regulated inflammation pathways^34^. Ye’s group showed that upconversion nanoparticles conjugated with curcumin as a photosensitizer to inhibit MRSA in lung under near infrared light^35^. These studies suggested that crucumin prevents *S. aureus* pneumonia maybe through multiple mechanisms^34^,^35^. Alough CUR exhibits a variety of pharmacological activities, the health benefits of CUR are limited by its poor bioavailability^36^. According to Kurien *et al*., the bioavailability and stability of heat-solubilized curcumin was significantly increased in experimental animals after oral administration^37^. However, further work is still needed to solve this issue^36^,^37^.

## Methods

### Bacteria and chemicals

*S. aureus* strains used in this study were a high-virulent clinical strain USA 300 (ATCC BAA-1717) and 8325-4, a high-level Hla-producing strain, and its cognate Hla-deficient strain DU 1090. CUR was commercially obtained from Chengdu Herbpurify CO., LTD (purity, 99.5%) (Chengdu, China) and dissolved in dimethyl sulfoxide (DMSO) to make stock solutions.

### Susceptibility testing and growth curve assay

The MICs of CUR were evaluated for *S. aureus* strains USA 300 and 8325-4 by using the broth microdilution method based on the Clinical and Laboratory Standards Institute (CLSI) guidelines.

For the growth curve assay, bacteria were grown in tryptic soy broth (TSB) at 37 °C to an OD_600 nm_ of 0.3 and then treated with various concentrations of CUR (0, 2, 4, 8 and 16 μg/ml). The cell growth at 37 °C with constant shaking under aerobic conditions was monitored by measuring the optical density at 600 nm (OD600) over 390 min.

### Hemolysis assay

*S. aureus* strains 8325-4 were cultured in TSB at 37 °C until the post-exponential growth phase was reached (OD_600 nm_ = 2.5) in the presence of indicated concentrations of CUR. Following centrifugation (10,000 × g, room temperature, 5 min), 100 μl of bacterial culture supernatants were mixed with 900 μl PBS buffer containing 3.0% defibrinated rabbit erythrocytes and the mixtures were incubated at 37 °C for 20 min. After centrifugation (5,500 × g, room temperature, 1 min), the absorption at 543 nm of supernatants was measured and the hemolysis percentage was calculated relative to the control culture, which was regarded as having 100% hemolytic activity.

To investigate the effect of CUR on hemolysis induced by wild type (WT)-Hla or its mutants, 100 μl of purified Hla (500 ng/ml) was pre-incubated in microtiter plates with graded concentrations of CUR at 37 °C for 15 min. Then, 100 μl (5 × 10^6 ^cells/ml) of defibrinated rabbit erythrocytes was added to each well for another incubation at 37 °C for 20 min. Lysis of the cells was determined as described above.

### Western blot assay

Bacterial culture supernatants as described above were subjected to 12% SDS-PAGE and the Hla proteins were then transferred to polyvinylidene fluoride membranes (Bio-Rad, Hercules, CA, USA). Membranes blocked by 5% non-fat dry milk in TBST were probed with a primary rabbit anti-Hla antibody (Sigma-Aldrich) diluted to 1:8000 and then with a horseradish peroxidase-conjugated secondary antibody (Sigma-Aldrich) diluted to 1:4000. The blots were developed with chemiluminescence detection Kit (ECL) (GE Healthcare, Buckinghamshire, UK).

### Live/dead and cytotoxicity assays

Bacteria were grown at 37 °C in TSB to an OD_600 nm_ of 0.5, and then 5 ml of the bacterial culture was centrifuged and resuspended in 10 ml of DMEM medium (Invitrogen, CA, USA). A549 human lung epithelial cells (ATCC CCL-185) were maintained in DMEM medium containing 10% fetal bovine serum (Invitrogen) at 37 °C with 5% carbon dioxide. Cells were seeded at approximately 2 × 10^5^ cells per well in 96-well plates and incubated overnight. Then, 100 μl *S. aureus* suspension was added to the wells with increasing concentrations of CUR (0, 2, 4, 8 and 16 μg/ml). Cell viability was measured by lactate dehydrogenase (LDH) release assay using Cytotoxicity Detection kit (LDH) (Roche, Basel, Switzerland) or live/dead cell staining with live/dead (green/red) reagent (Invitrogen) following 7 h incubation at 37 °C as described previously.

### Mouse model of intranasal lung infection

C57BL/6J mice were supplied by the Jilin University Experimental Animal Center (Changchun, China). Mice used for the experiments were housed and handled in accordance with the guidelines established by Jilin University. These studies were reviewed and approved by the Animal Welfare and Research Ethics Committee of Jilin University.

Bacteria were grown at 37 °C in TSB to an OD_600 nm_ of 0.5, pelleted, resuspended in PBS and quantified by visible spectrometry readings at 600 nm. For the lung infection, 6 to 8-week-old female C57BL/6J mice were nasally infected with 2 × 10^8^ CFUs of *S. aureus* after anaesthetized with ketamine and xylazine. The *S. aureus* infected mice were subcutaneously administered 100 mg/kg of CUR 2 h after infection and then at 8 h intervals. Infected mice injected with DMSO on the same schedule were served as controls and each experimental group contained 20 mice. The mortality rate of infected mice was monitored daily. For histopathological analysis, the lungs were fixed in 10% formalin, dehydrated in an alcoholxylene series, embedded in paraffin, stained with hematoxylin-eosin and analyzed by light microscopy.

### Molecular modeling

The standard docking process was performed for Hla and the drug, and the detailed procedure was reported in our previous literates^5^,^18^. Based on the 3D structure of the complex obtained by molecular docking, the molecular simulations for the ligand with WT-Hla and mutants were calculated by using Gromacs 4.5.1 software package^38^. To insight into the interaction mechanism between the drug and the protein, the MM-GBSA method was used to calculate the binding free energy between the ligand with protein (including the ligand-residue interaction decomposition)^39^,^40^.

### Mutagenesis of the Hla protein

The recombinant plasmids encoding WT-Hla and its two mutants, Q89A-Hla and K163A-Hla, were constructed as described in our previous work^5^,^18^. The mutagenic primers for Q89A-Hla were 5′-GCCTTTAAGGTACAGTTGGCGCTACCTGATAATGAAGTAG-3′ (forward) and 5′-CTACTTCATTATCAGGTAGCGCCAACTGTACCTTAAAGGC-3′ (reverse), and those for K163A-Hla were 5′-GAGAGCCCAACTGATGCGAAAGTAGGCTGGAAAG-3′ (forward) and 5′-CTTTCCAGCCTACTTTCGCATCAGTTGGGCTCTC-3′ (reverse). After transforming the recombinant plasmids into *Escherichia coli* strain BL21(DE3) (Novagen), the soluble Hla proteins were obtained from induced *E. coli* cells under the appropriate conditions for high-level expression and purification of recombinant Hla protein described by Qiu *et al*.^5^.

### Fluorescence-quenching assay

In our paper, fluorescence quenching can be described by the following Scatchard equation: *r*/*D*_*f*_  = *nK* − *rK*, where *r* is the ligand amount of substance per mole of protein binding (*r *≈ *ΔF*/*F*_*0*_), *D*_*f*_ is the free concentration of CUR, *K* is the binding constant and n is the number of binding sites. In this system, due to the CUR concentration being far greater than the concentration of protein; the *D*_*f*_ is replaced by the total CUR concentration, *Q*. According to experimental results, the linear fitting plots of *r*/*D*_*f*_ vs. *r* between CUR and WT-Hla or its two mutants (K163A-Hla and Q89A-Hla) can be made in Fig. 6 and based on the plots the values of *K* and *n* can be obtained. In this work, Hla can act as the Fluorescence and CUR act as the quencher. Fluorescence spectrofluorimetry measurements were carried out using a Horiba Jobin-Yvon Fluorolog 3-221 spectrofluorometer (Horiba Jobin-Yvon, Edison, NJ). The measurements were acquired using a 280-nm excitation wavelength with a 5-nm band-pass and a 345-nm emission wavelength with a 10-nm band-pass.

### Oligomerization assay

For the oligomerization assay, 20 μg of purified recombinant protein (WT-Hla, Q89A-Hla or K163A-Hla) treated with or without CUR was incubated with 5 mM deoxycholate at 22 °C for 25 min. Next, the mixtures were mixed with 5 × loading buffer without β-mercaptoethanol and incubated at 55 °C for 10 min. 25 μl of reaction mixtures were loaded to 10% SDS-PAGE followed by western blot analysis.

### Statistical analysis

The statistical significance of the mortality studies was assessed using Fisher’s exact test; the significance of hemolysis, in terms of the LDH release assay results, was calculated using the two-tailed Student’s t-test.

## Additional Information

**How to cite this article**: Wang, J. *et al*. Curcumin protects mice from *Staphylococcus aureus* pneumonia by interfering with the self-assembly process of α-hemolysin. *Sci. Rep*. **6**, 28254; doi: 10.1038/srep28254 (2016).

## Supplementary Material

Supplementary Information
