# Supplementary material for: Curcumin protects mice from Staphylococcus aureus pneumonia by interfering with the self-assembly process of α-hemolysin
Source: Sci Rep. 2016 Jun 27;6:28254. doi: 10.1038/srep28254 (PMC4921848; doi:10.1038/srep28254)
Supplement: Supplementary Information [file srep28254-s1.doc]

**Curcumin protects mice from *Staphylococcus aureus* pneumonia by interfering with the self-assembly process of α-hemolysin**

Jianfeng Wang, Xuan Zhou, Wenhua Li, Xuming Deng, Yanhong Deng*, Xiaodi Niu*

Key Laboratory of Zoonosis, Ministry of Education, Institute of Zoonosis, and Department of Food Quality and Safety, College of Veterinary Medicine, Jilin University, Changchun, China

*Correspondence and requests for materials should be addressed to X.D.N. (niuxd@jlu.edu.cn) or Y.H.D. (yanhong7@sohu.com).

**Supplementary results**

**Supplementary Table 1** Calculated energy components, binding free energy (kcal/mol) of CUR binding to active site of Hla.

| **Energy components (kcal/mol)** | **WT-Hla** | **K163A-Hla** | **Q89A-Hla** |
| --- | --- | --- | --- |
| *ΔE_ele_*  *ΔE_vdw_*  *ΔE_MM_*  *ΔG_ele,sol_*  *ΔG_nonpolar,sol_*  *ΔG_sol_*  *ΔG_ele,sol_* + *ΔE_ele_*  *ΔG_nonpolar_* + *ΔE_vdw_*  *ΔG_total_*  *-TΔS*  *ΔG_bind_* | -15.96 ± 3.08  -24.22 ± 1.86  -40.19 ± 3.12  -3.48 ± 0.14  27.14 ± 3.12  23.66 ± 3.09  -19.44 ± 3.11  2.92 ± 2.81  -16.52 ± 2.11  2.95 ± 1.71  -13.57 ± 1.34 | -27.09 ± 3.09  -22.70 ± 2.55  -49.79 ± 4.25  -4.19 ± 0.14  40.83 ± 3.55  36.64 ± 3.12  -31.28 ± 3.16  18.13 ± 2.76  -13.15 ± 1.69  2.87 ± 1.54  -10.28 ± 1.17 | -2.06 ± 1.06  -20.91 ± 2.06  -22.98 ± 3.16  -3.71 ± 0.23  16.16 ± 3.14  12.45 ± 2.92  -5.77 ± 1.14  -4.75 ± 1.58  -10.52 ± 1.71  3.09 ± 1.48  -7.43 ± 1.56 |

**Supplementary Table 2 Values of the binding constants (*K_A_*) and the number of binding sites (n) of Hla-CUR systems based on the fluorescence-quenching method.**

|  | **WT-Hla** | **K163A-Hla** | **Q89A-Hla** |
| --- | --- | --- | --- |
| *K*_A_ (1×10^4^) L·mol^-1^  n | 3.292 ± 1.32  1.1531 | 2.109 ± 1.24  1.1645 | 1.986 ± 1.21  0.9996 |
